# Allelic Variation of the *MMP3* Promoter Affects Transcription Activity through the Transcription Factor C-MYB in Human Brain Arteriovenous Malformations

**DOI:** 10.1371/journal.pone.0057958

**Published:** 2013-03-04

**Authors:** Cong Huai, Jianping Song, Zengyi Ma, Xuanfeng Qin, Peiliang Li, Hongyan Chen, Fan Zhao, Daru Lu, Donglei Song, Ying Mao, Xiao Song, Yao Zhao

**Affiliations:** 1 State Key Laboratory of Genetic Engineering, Fudan-VARI Genetic Epidemiology Center and MOE Key Laboratory of Contemporary Anthropology, School of Life Sciences and Institutes for Biomedical Sciences, Fudan University, Shanghai, China; 2 Department of Neurosurgery, Huashan Hospital, Fudan University, Shanghai, China; CNRS UMR7275, France

## Abstract

MMPs comprise a family of proteolytic enzymes that degrade pericellular substances, which may result in the destabilization of vessels and related to the development of brain arteriovenous malformations (BAVM). *MMP3* is a key member of this family, overexpressed in BAVM tissues, and a single nucleotide polymorphism within *MMP3*, −709A>G (rs522616), is significantly associated with the risk of BAVM. In this study, we aimed to investigate the mechanism through which the polymorphism rs522616 regulates the expression of *MMP3*. Our results showed that −709A led to a over 2-fold higher transcriptional activity compared with the G allele (*P*<0.05) and this transcriptional activity can be depressed by co-transfecting cells with competitive DNA fragments containing −709A but not −709G. Bioinformatics analyses suggested that the transcription factor C-MYB might bind to the area around rs522616. Overexpressed C-MYB significantly increased the transcriptional activity of −709A compared with −709G or controls that did not overexpress *c-myb* (*P*<0.01) in HEK293 and HUVEC cells. ChIP assays indicated that C-MYB bound to the SNP region in the two cell lines and three BAVM tissue samples. Together, these data indicated that C-MYB can bind to the −709A allele of the *MMP3* promoter, activate its transcription and lead to a higher expression of this gene. This novel hypothesis, supported by molecular evidence, explains how this SNP affects *MMP3* promoter function and results in a risk of BAVM development.

## Introduction

Brain arteriovenous malformations (BAVM) are relatively infrequent but important sources of spontaneous intracranial hemorrhage (ICH) and may cause a life-threatening ICH in 2–6% of cases annually, which would result in high neurological morbidity in young adults [1], [2]. Although most discovered BAVMs can be treated by some combination of surgical resection, endovascular embolization and radiosurgery, patients with typical BAVMs of high Spetzler-Martin grade are still facing huge therapeutic risks.

The genesis of AVMs is still unclear, but several gene mutations, such as *ALK-1* (Activin-like kinase receptor 1) variants, have been associated with a risk of sporadic BAVM [3], [4], [5]. BAVMs are often presumed to be congenital, but there is little direct evidence to support this idea. However, recent studies indicate that BAVMs can grow or regress after birth due to active angiogenesis [6], [7], [8], [9]. Because post-natal growth is possible and even likely, one plausible basis for therapy would be further slow this already very slow growth over time.

Based on the existing literature, we hypothesized that BAVMs could arise congenitally due to specific gene mutations and could also be stimulated by some post-natal events. The inciting events might include subclinical injury from otherwise unremarkable episodes of trauma, infection, inflammation, irradiation or compression. Then, VEGF and other inflammation factors activate angiogenesis, followed by the excessive degradation of the vascular matrix by matrix metalloproteinase (MMPs) [4], [5]. MMPs comprise a family of proteolytic enzymes that degrade extracellular matrix (ECM) proteins, cell surface molecules and other pericellular substances, which may result in the destabilization of vessels [5], [7], [10], [11]. This is a critical step in further angiogenesis and vascular remodeling. It appears that histological prototype of BAVM, which is called vascular dysplasia is developed after this event [12].

The basic morphology of a mature BAVM is a vascular mass, called the nidus, which is a complex tangle of abnormal, dilated channels that are not clearly arterial or venous, with intervening gliosis that directly shunts blood between the arterial and venous circulations without a true capillary bed [13]. Thus, we suggest that abnormal vascular remodeling, mainly stimulated by MMPs, is more important in BAVM development. The increased expression of MMPs in BAVM tissues have been confirmed [14], [15], and MMP3 is an crucial activator of a number of pro-MMPs [14], [15]. In vitro, endothelial cell proliferation and migration could be affected by elevated MMP3 [16].

In our previous case-control study, we found that a single nucleotide polymorphism rs522616 A>G (−709 A>G) variant of the *MMP3* promoter was significantly associated with BAVM in a Chinese Han population (*P* = 0.02). Logistic regression analysis revealed that the variant genotype G was associated with a significantly decreased risk of BAVM (adjusted odds ratio = 0.68 for the AG+GG vs the AA genotype). This finding indicated that the *MMP3* rs522616 polymorphism may contribute to the etiology of sporadic BAVM in the Chinese Han population [11]. However, the relationship between the rs522616 polymorphisms and the expression of *MMP3* remains unclear. In this study, we compared the transcriptional activities of the *MMP3* promoters with A or G alleles to determine the molecular biological effects of the *MMP3* rs522616 polymorphism.

## Materials and Methods

### Cell Culture and Tissue Samples Collection

HEK293 cells and HUVEC cells were obtained from the Type Culture Collection of the Chinese Academy of Sciences, Shanghai, China. All cells were cultured in Dulbecco’s modified Eagle’s medium (DMEM; GIBCO, USA), with 10% fetal bovine serum (FBS; GIBCO, USA), 100 U/mL penicillin, and 100 µg/mL streptomycin (Sigma, USA) and maintained at 37°C in 5% CO_2_.

The tissue samples of BAVM and adjacent tissues (normal control) were obtained from 3 patients who underwent open BAVM resection surgery between 2010 to 2011 at Huashan Hospital, Shanghai, China, 2 male and 1 female patients, age from 26–50 years old. The obtained tissue specimens were immediately snap frozen in liquid nitrogen and then stored at −80°C. The study was approved by the ethical review committee of Huashan Hospital, Fudan University. All patients were provided informed consent and signed to agree with the use of their tissues for this original human work.

### RNA Extraction, cDNA Synthesis and Real-time PCR

RNAs of the three BAVM and control samples were isolated by Trizol (Invitrogen, CA), and all cDNAs were synthesized using ReverTra Ace® qPCR RT Kit (Toyobo, Japan) from 1 µg RNA. *MMP3* expression was quantitated by real-time PCR with the cDNAs from these tissues using SYBR® Green Realtime PCR Master Mix (Toyobo, Japan), and was performed with an ABI 7900 instrument (Life technology, USA). The cycling conditions were 95°C for 3 min followed by 40 two-step cycles- 95°C for 15 s, 60°C for 30 s. The quantitative amplification of every sample was performed triplicate, and the mean Ct values were used to calculate expression levels of *MMP3*, using the relative standard curve method. Glyceraldehyde-3-phosphate dehydrogenase (*GAPDH*) was used as the endogenous control to obtain normalized values. The sequences of the PCR oligonucleotide primers were: *MMP3* forward 5′-TGAGTCAATCCCTGGAAAGTC- 3′, reverse 5′-TGAGTCAATCCCTGGAAA GTC-3′; *GAPDH* forward 5′-GAAGGTGAAGGTCGGAGTC-3′, reverse 5′- GAAGATGGT GATGGGATTTC-3′.

### HE Staining and Immunohistochemistry

HE staining and immunohistochemistry were performed with all three paraffinembedded BAVM tissues and normal tissues to test the expression of MMP3 in BAVM. The routine HE staining was performed to detect the histological character of individual specimen. Immunohistochemical staining was carried out by Envision technique using polyclonal antibody to MMP3 (Abcam, ab53015, USA) with 1∶500 dilution.

### Reporter Plasmids Construction and Transcription Factor Prediction

**Figure 1:**
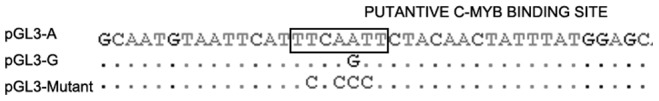
sequencing results of different p*MMP3*-luciferase plasmids.

To compare the transcriptional activity of promoters with different allele, reporter plasmids pGL3-A-p*MMP3* (pGL3-A)/pGL3-G-p*MMP3* (pGL3-G) were generated by cloning promoter segments of the human *MMP3* gene from 1084 bp upstream of the initiation sequence to the transcription start site (−1084∼−1), including the single nucleotide polymerase rs522616 A/G at −709, into the luciferase reporter plasmid pGL3-Basic (Promega, Sweden). The −709A promoter segment was first amplified by PCR using the High Fidelity DNA polymerase KOD-Plus (Toyobo, Japan) with the upstream primer p1 and downstream primer p2 (the sequences of all primers are shown in Table 1.). *Kpn*I and *Xho*I recognition sequences of were added to the 5′ -end of the PCR fragment to be ligated into the vector. pGL3-G and pGL3-mutant plasmids were generated by site-directed mutagenesis PCR using the primer pairs p1/p3, p2/p4 and p1/p5, p2/p6, respectively. The sequencing results for all plasmids are shown in Fig. 1.

**Table 1:** Primers used for construct p*GL3*-p*MMP3*-luciferase plasmid.

Name	Sequence
P1	GGCAGGTGGCA GAGGACT
P2	TTCCACTGGCTTTACTTAGCTCTATG
P3	GTTGTAGAATTGAAATGAATTACATTGC
P4	TCATTTCAATTCTACAACTATTTATGGAG
P5	GCAATGTAATTCATTCCCCCTCTACAACTATTTATG
P6	CATAAATAGTTGTAGAGGGGGAATGAATTACATTGC

The web-based transcription factor site prediction programs TFSEARCH (http://www.cbrc.jp/research/db/TFSEARCH.html) and TESS (http://www.cbil.upenn.edu/cgi-bin/tess/tess) were used to identify the transcription factors that might bind to the −709 A/G region of the *MMP3* promoter. Because these results suggested that C-MYB may bind to this area, the *c-myb* expression vector pcDNA3.1-*c-myb* was generated by cloning the cDNA into pcDNA3.1 with *Eco*RI and *Bam*HI. All constructs were confirmed by sequencing.

### Transient Transfection and Luciferase Assay

The transcriptional activities of different sets of *MMP3* promoters were determined by measuring luciferase reporter gene expression. The Translipofect liposome kit (Tiangen Biotechnology, China) was used to transfect the vectors into HEK293 and HUVEC cells. Briefly, 1×10^5^ cells were plated into each well of a 24 well plate in 500 µL medium for 24 hours, allowing them to reach 90% confluence at the time of transfection. Diluted plasmid (800 ng constructed plasmids with 30 ng pLV-*Renilla* luciferase plasmid) was mixed with diluted liposome (2 µL) in 100 µL Opti-MEM I medium. After incubating for 20 minutes at room temperature, 100 µL of this complex was added to each well containing cells and 400 µL FBS-free medium. The medium was changed to fresh DMEM with 10% FBS after 6 hours.

After co-cultured for 24 hours, cells were washed twice with PBS and extracted in 200 µL of 1× passive lysis buffer. Luciferase activity was measured with the Dual-Luciferase Reporter Assay System on GLOMAX™ 96 microplate luminometer (Promega, Sweden). Promoter activities were expressed as the ratio of firefly luciferase to *Renilla* luciferase activities.

In the competition experiment, 100-fold excess DNA fragments of *MMP3* promoter fragment containing −709A or −709G allele, or a control portion with 5 bases mutated to C at the SNP section was co-transfected with firefly pGL3-A and pLV-*Renilla* luciferase plasmid to HEK293 cell, to test the differential binding affinity to transcription factor of the two alleles. The sequences of each competitors were as follows: A competitor 5′-AAATCCGGTAAGCA ATGTAATTCATTTCAATTCTACAACT-3′, G competitor 5′- AAATCCGGTAAGCAATGT AATTCATTTCAGTTCTACAACT -3′, and control competitor 5′- AAATCCGGTAAGCAA TGTAATTCACCCCCCTCTACAACT -3′. In the overexpression experiment, 400 ng of pcDNA3.1-*c-myb* or the vector control pcDNA3.1 was co-transfected with these two reporters to determine whether the up regulation of C-MYB had differential effects on A or G allele.

### Chromatin Immunopreciption (ChIP) Assay

ChIP assays were performed using a chromatin immunoprecipitation assay kit (Beyotime Biotechnology, China). Briefly, 6×10^6^ HEK293 or HUVEC cells or 100 mg homogenized tissue samples were treated with 1% formaldehyde for 20 min at 37°C to cross-link the protein to the DNA. Cells or tissues were then suspended in SDS lysis buffer. The lysates were sonicated with thirty cycles of 10 s sonication and 50 s idle time to shear the DNA into fragments of 200–1000 base pairs using Ultrasonic Cell Disruptor JY92-II (Xinzhi Biotechnology, China). One-third supernatant was set aside as a positive control, and the remaining samples were diluted 5-fold in ChIP dilution buffer and precleared with salmon sperm DNA/protein A+G agarose. Half of the diluted samples received no antibody (NA) and the other half was incubated with 2 µL anti-C-MYB antibody (Abcam, ab45150, USA) overnight at 4°C. All samples were precipitated with a salmon sperm protein A+G agarose slurry and washed extensively. The protein-DNA crosslinks were reversed by heating the samples at 65°C for 4 hrs with 0.2 mol/L NaCl. All the samples and positive controls were phenol/chloroform-extracted, ethanol-precipitated and then used for PCR with the forward primer 5′- CTGTTCTTCAACTTCAAAGCATCTG -3′ and reverse primer 5′- CCACGTAG CTGCTCCATAAAT -3′. The primers for negative control PCR was forward: 5′- TGGTCCA TACCCTCAAGTAGCT -3′, reverse: 5′- CCTGAAAGATGTTTCCCAAGTC -3′.

### Statistical Analysis

Statistical analysis was performed using SPSS 15.0 and Microsoft Excel 2007. The Student’s t-test was used for statistical comparisons between groups. All of the calculated *P*-values were two-tailed, and a level of *P*<0.05 was considered to be statistically significant.

## Results

### MMP3 Overexpressed and Positive Immunohistochemical Staining in the Endothelial Cell/peri–endothelial Cell Layer of BAVMs

**Figure 2:**
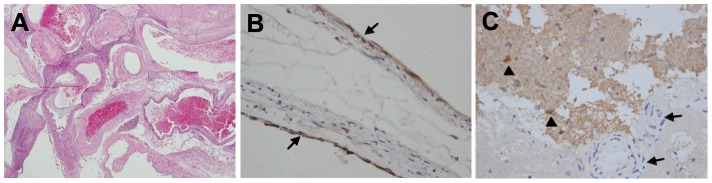
MMP3 was overexpressed in BAVM. *A*, HE staining (magnification×40) verified a typical histological character of BAVM. *B*, the immunohistochemical staining showed that MMP3 was localized mainly in the endothelial cell layer of BAVMs (arrows, magnification×200). *C*, MMP3 was strongly immunostained in neurons (triangle) but not in vascular structures (arrows, magnification×400).

To detect the mRNA and protein expression level of MMP3 in BAVM, real-time PCR and immuohistochemical staining were performed in the AVM and adjacent tissues(control) from the three patients. In real-time PCR, the mean Ct values of the three control tissues were set as reference (1.0). Compared with the reference, *MMP3* expression in BAVM samples increased to 4.1 fold, 18.5 fold and 33.6 fold respectively. HE staining showed the typical histological character of BAVM (Fig. 2A) and the immunohistochemical staining demonstrated that MMP3 was localized mainly in the endothelial cell/peri–endothelial cell layer of BAVMs (Fig. 2B). However, immunohistochemistry on control brain samples showed that MMP3 was strongly expressed in neurons but not in vascular structures (Fig. 2C), which confirmed that the expression of MMP3 was up-regulated in BAVMs.

### The −709 A>G Polymorphism in the MMP3 Promoter Resulted in Decreased Transcriptional Activity

**Figure 3:**
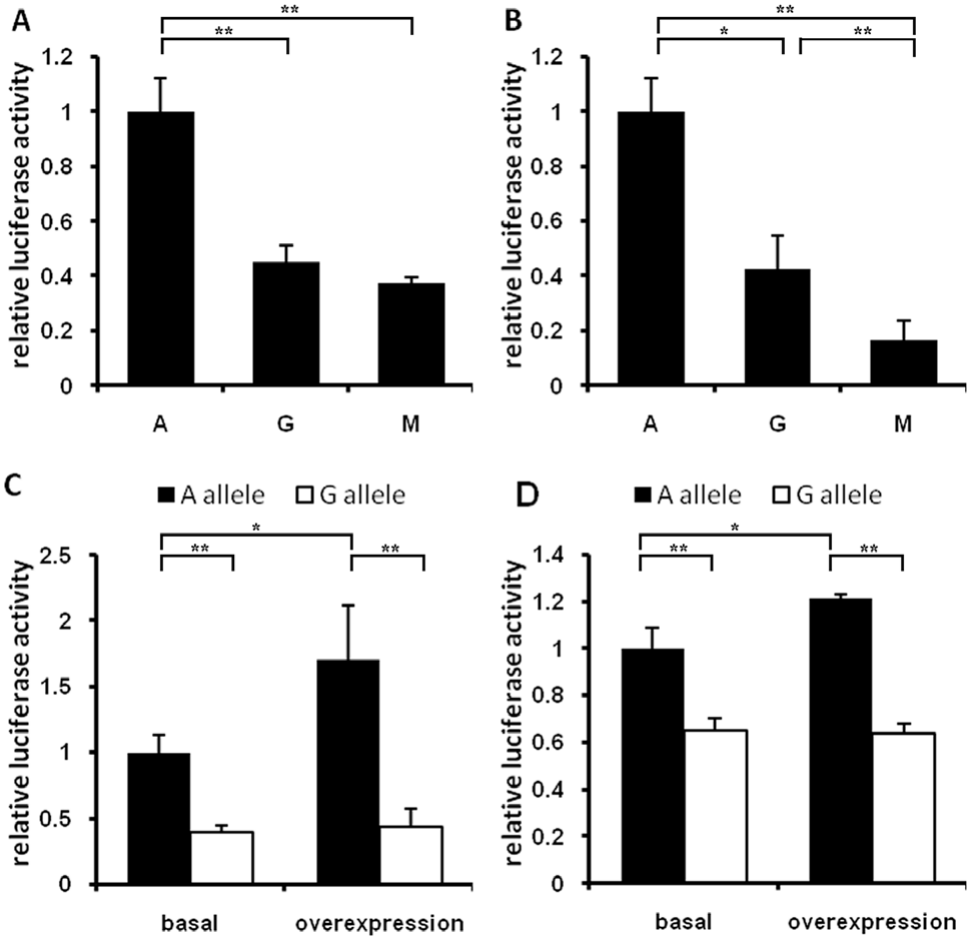
*MMP3*−709A>G variant resulted in a decreased transcriptional activity and C-MYB can activate −709A. *A,B* luciferase activities in HEK293 cells and HUVEC cells caused by transfected with pGL3-A, pGL3-G or mutant reporter respectively, normalized by *Renilla* luciferase. *C* and *D* pcDNA3.1 (basal) or pcDNA3.1-myb (overexpression) was co-transfected with luciferase reporter constructs A or G, show the normalized luciferase activities in C-MYB basal and overexpression level. Statistical analysis was performed by a two-tailed *t* test.

To determine whether the −709A>G polymorphism affects *MMP3* gene expression, we measured the transcriptional activity of each *MMP3* promoter with a luciferase assay, transiently transfecting the pGL3-A/G reporter plasmids into HEK293 cells and HUVEC cells. The luciferase activity in each transfection was normalized to that of the pGL3-A vector as a reference (1.0). In HEK293 cells, the −709G promoter resulted in a 2.21-fold (*P*<0.01) decrease in expression compared with −709A (Fig. 3A); this ratio was 2.36 in HUVEC cells (*P*<0.05, Fig. 3B). It suggested that −709 A is associated with a higher transcriptional activity of the *MMP3* promoter than variant G allele.

### The −709A Promoter had a Stronger Transcription Factor Binding Affinity in vivo

To investigate whether the differential expression of the A and G alleles were caused by differential binding to some transcription factor, a 5-bases mutant reporter (pGL3-mutant) was also transfected into the two cell lines. The luciferase activity of the A allele was notably up-regulated compared with the pGL3-mutant construct both in HEK293 cells and HUVEC cells with 2.67-fold and 5.89-fold changes, respectively (*P*<0.01, Fig. 3A, 3B). There was no statistically significant difference in expression between the G allele and the pGL3-mutant in HEK293 cells, but the expression of G allele was significantly higher in HUVEC cells (*P<*0.01). This implied that the sequence near this SNP may include a binding site for one or more transcriptional factors and that the presence of A or G at this position may change the binding affinity of those factors.

### A Competitive DNA Fragment Containing the A Allele Decreased the Binding with Transcription Factor

**Figure 4:**
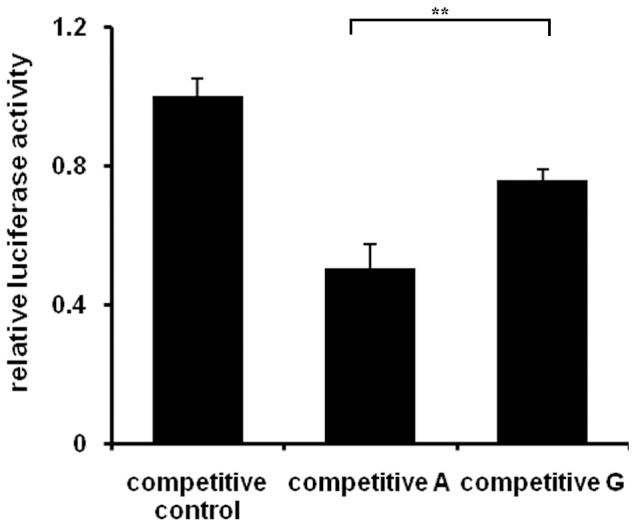
Competitive DNA fragment containing the A allele decreased MMP3-A’s binding with transcription factor. 100 fold excess competitive *MMP3* promoter portions containing A or G allele or control potions with 5 baseds mutations at the SNP section were co-transfected with pGL3-A reporter to HEK293 cell respectively and normalized by *Renilla* luciferase. The luciferase activity in control group was set as reference (1.0). Statistical analysis was performed by a two-tailed *t* test.

To compare the transcription factor binding affinity, 100 fold excess DNA fragments of the *MMP3* promoter bearing the A or G allele were co-transfected with the −709A luciferase reporter plasmid into HEK293 cells as competitors and control fragments which mutated 5 bases at the SNP section was set as reference (1.0). Compared with the reference, the co-transfected A portion led to a greater decrease in luciferase activity (to 0.503-fold) than did the G portion (to 0.756-fold, *P*<0.01, Fig. 4). It confirmed that the A allele has a greater binding affinity to transcription factors in vivo.

### Overexpression of the Transcription Factor C-MYB Increased the Transcriptional Activity of the −709A Promoter

According to predictions made by the of TESS program, the transcription factor C-MYB may bind to the “TTCAATT” sequence from −713 to −707 in the *MMP3* promoter, which involves −709A (Fig. 1). However, the −709 A>G polymorphism changes this sequence to “TTCAGTT”, which may destroy the binding site with C-MYB or lead to a weaker affinity.

A *c-myb* expression plasmid was co-transfected with the pGL3-A vector or pGL3-G vector respectively into HEK293 cells and HUVEC cells. Compared with the *c-myb* baseline expression group, the luciferase activity of pGL3-A was significantly increased in the overexpression group (*P*<0.05), by a 1.70-fold in HEK293 cells and 1.21-fold in HUVEC cells (*P<*0.05, Fig. 3C, 3D), indicating that C-MYB enhanced the transcriptional activity of the promoter with the A allele. However, there was no significant difference in the transcription activity of pGL3-G both in HEK293 cells and HUVEC cells (*P* = 0.713 and 0.618, respectively). These finding implied that the added *c-myb* in cell ascended the difference of transcriptional activity of −709A/G *MMP3* promoter, and expression of *c-myb* mediated the difference in the transcriptional activity of the −709A/G *MMP3* promoter.

### C-MYB Binds to the MMP3 Promoter in vivo

**Figure 5:**
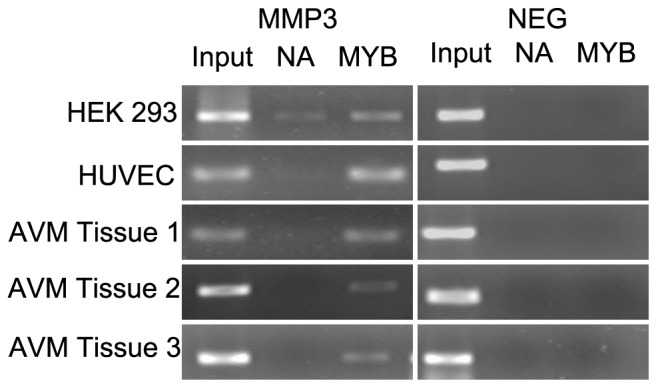
C-MYB binded to the *MMP3* promoter in vivo. ChIP assay carried out using antibody against C-MYB (or no antibody as negative control) in 2 cell lines and 3 AVM tissue samples. PCR products “MMP3” represent the amplicon amplified from MMP3 promoter from −643 to −772. PCR products “NEG” represent the amplicon amplified from the intron of the gene. Input, positive control using total genomic DNA as template; MYB, PCR products amplified from the ChIP samples mediated by anti-c-myb antibody; NA, PCR products using ChIP samples by non-antibody control.

To investigate whether C-MYB bound directly to the *MMP3* promoter, ChIP assays were carried out in HEK293 cells, HUVEC cells and 3 AVM tissue samples. PCR analysis using a specific primer targeting the *MMP3* promoter from −643 to −772 revealed a single PCR amplicon, which was confirmed by sequencing. The negative control targeting a *MMP3* intron downstream in the gene. A weaker band was detected when no antibody was present in a ChIP assay using HEK293 cells, but no band was detected in HUVEC cells or the 3 tissue samples (Fig. 5). Moreover, the sequencing results of PCR amplicons from ChIP products confirmed the binding of *MMP3* promoter with C-MYB, and showed a increased proportion of −709A in MYB group compared with the input group. These findings suggested that C-MYB can bind directly to the SNP −709 A>G region of the *MMP3* promoter in vivo and have a high infinity with −709A.

## Discussion

Although the cause of BAVM is unknown, lines of evidence indicate that BAVM may occur because of pressure and damage to blood vessel tissue. With the weakening of the vessel walls, the vessel structure changes, and blood may leak into the brain or surrounding tissues. The treatment of BAVM includes open surgery, interventional therapy and radiotherapy, but for large and high Spetzler-grade BAVM, few treatments are available [17]. It is indeed needed to get clear how BAVMs generate and develop.

Matrix metalloproteinases (MMPs) are a family of zinc-dependent proteolytic enzymes that degrade ECM and basement membrane barriers to remodel and maintain the pericellular environment, [10] which may result in the destabilization of vessels and lead to angiogenesis [18], [19], [20]. MMPs are dyregulated in almost every human cancer [19].

*MMP3* is a key member of the MMP family, which participates in regulating the accumulation of ECM and activating the other MMPs (such as MMP2 and MMP9) [21]. It has been reported to release cell surface molecules, including E-cadherin [22], promote mammary carcinogenesis [23] and is related to several diseases that are characterized by unstable vascular and matrix scaffolds. In this study, we found that the expression of MMP3 increased in vessel endothelial cells and adventitia in BAVM tissues by immunohistochemical staining, which implies that the overexpression of *MMP3* affects the tumorigenesis of BAVM.

Further, we detected differential transcriptional activity of the *MMP3* promoter caused by −709 polymorphism, finding that the minor allele G induced a lower transcriptional activity and lead to a decreased expression of *MMP3* than did the major allele A. This result is consistent with our previous finding in an epidemiology case-control study that variant allele −709G was significantly associated with a decreased risk of developing BAVM [19]. The overexpression of MMP3 may be a risk factor for BAVM tumorigenesis, while decreased *MMP3* expression could be a protective factor.

The risk of tumor development conferred by the A allele has been reported in several different types of cancers and in different ethnic populations. In addition to its association with BAVM mentioned above, the A allele was also found to increase the incidence of gliomas (HR = 2.36, *P* = 0.022), esophageal carcinoma and lung cancer in the Chinese Han population [24], [25]. Moreover, in US datasets, rs522616A was identified as an ovarian cancer susceptibility “hot-spot” [26] and was significantly related to the development of chronic periodontitis. However, all of these results were reported in simple gene association studies, and none provided any experimental evidence of the effects of this allele. The findings presented in this study offer a mechanistic explanation for the risk presented by −709A and provide molecular evidence that the SNP in *MMP3* contributes to the etiology of BAVM.

Furthermore, we clarified how the −709A allele of the *MMP3* promoter up-regulated the gene expression. Bioinformatics analyses suggested that the transcription factor C-MYB might bind to the promoter containing the A allele allele but not containing the G allele, and it was confirmed to bind to the SNP region both in cell lines and in AVM samples in vivo. As *c-myb* is a famous oncogene that is overexpressed in most cancers or pretumorous cells [27], [28], our finding that *c-myb* increased the expression driven by the A allele promoter but not the G allele promoter implies that *c-myb* may be one important factor that interacts with rs522616 to regulate the *MMP3* expression.

However, there are also some contradictory findings in epidemiology studies. For example, in eastern India, the *MMP3* −709GG genotype conferred a risk for gastric cancer development [29]. It is clear that C-MYB cannot be the only transcription factor that interacts with the rs522616 region; other transcription factors such as TST-1 (identified by *TF-search* prediction), C/EBP and Cre-BP1 [25] may also bind to neighboring locations. In particular, C/EBP, has a target sequence within this region and it is a CCAAT enhancer factor, which may help elevate the gene expression. C/EBP is also thought to be related to tumor invasion and migration [30]. For example, elafin is activated by C/EBP β in breast cancer cells, promoting cell migration [31]. The complex regulation of the *MMP3* promoter may also explain the differences in reporter activity between pGL3-mutant and pGL3-G in HUVEC cells observed here and the opposite findings in others’ research.

### Conclusions

we carried out this study to investigate the effects of the −709 A/G polymorphism on the transcriptional activity of *MMP3* promoter. We found that the A allele notably increased the expression of *MMP3* compared with the G allele. A competition assay indicated that the addition of excess A fragment significantly decreased the luciferase activity of the reporter compared to the addition of excess G fragment. We also predicted and confirmed that C-MYB can bind to the SNP region and activate only the A allele promoter. Meanwhile, other studies predicted that other transcription factors might bind around this region, but it is not clear whether these transcription factors cooperate in regulating *MMP3* gene expression. More experiments are needed to fully elucidate how the SNP rs522616 affects MMP3 expression and the development of BAVMs.
